# Repositioned versus exchanged flanged intraocular lens fixation for intraocular lens dislocation

**DOI:** 10.1038/s41598-024-54694-6

**Published:** 2024-03-14

**Authors:** Yong Koo Kang, Dong Ho Park, Gahyung Ryu, Hong Kyun Kim, Dong Hyun Kim, Jae Rock Do

**Affiliations:** 1 Department of Ophthalmology, School of Medicine, Kyungpook National University, Kyungpook National University Hospital, 130 Dongdeok-Ro, Jung-Gu, Daegu, 41944 Republic of Korea; 2 Nune Eye Hospital, Seoul, Republic of Korea

**Keywords:** Refractive errors, Retinal diseases

## Abstract

This study aimed to compare the outcomes of flanged intraocular lens (IOL) fixation with new IOL exchange after dislocated IOL removal and repositioned dislocated IOL in patients with IOL dislocation. Eighty-nine eyes that underwent flanged IOL fixation were retrospectively included, with 51 eyes in the exchanged IOL group and 38 eyes in the repositioned IOL group. In both groups, best-corrected visual acuity (BCVA) improved at 1, 3, 6, and 12 months postoperatively and did not differ between the two groups at any of these time points. However, at 1 week postoperatively, BCVA in the repositioned IOL group improved compared with baseline, whereas that in the exchanged IOL group did not. Moreover, there were lesser changes in the corneal endothelial cell density (ECD) and corneal astigmatism in the repositioned IOL group than in the exchanged IOL group. The IOL positions, including IOL tilt and IOL decentration, were not different between the groups. Flanged IOL fixation with new IOL exchange and with repositioned dislocated IOL for patients with IOL dislocation had similar visual outcomes and IOL position. However, the latter had a smaller corneal ECD decrease and astigmatic change. This technique was effective in treating IOL dislocation while minimizing corneal injury.

## Introduction

Intraocular lens (IOL) dislocation is one of the most common complications requiring surgical treatment following cataract surgery^1^. With improvements in surgical techniques, the occurrence of other complications decreased overtime; however, the incidence of IOL dislocation increased^1,2^. Thus, the use of secondary IOL implantation for managing IOL dislocation is predicted to further increase.

Secondary IOL implantation involves various surgical techniques, including iris fixation^3^, retropupillary iris claw IOL implantation^4^, four-haptic IOL fixation^5^, and sutured scleral fixation^6^. Recently, Yamane et al.^7^ reported a new surgical technique, namely, sutureless flanged IOL fixation, in which two IOL haptics are fixed to the sclera by attaching the haptic ends to the flange with cauterization. Owing to its shorter operating time and similar effective visual outcomes to conventional sutured scleral fixation^8^, this technique has been widely employed.

Typical secondary IOL implantation, including flanged IOL fixation, was performed after dislocated IOL removal through the corneal incision site. This procedure may damage corneal endothelial cells. Severe reduction of corneal endothelial cell density (ECD) following intraocular surgery may cause serious complications such as bullous keratopathy. However, if the flanged IOL fixation is used to reuse a dislocated IOL, the needle and haptic of the dislocated IOL can be directly docked in the vitreous cavity. Therefore, in this technique, IOL removal, corneal incision, and any additional manipulation in the anterior chamber are not required. Thus, flanged IOL fixation with repositioned dislocated IOL is assumed to be less traumatic and associated with less corneal changes. In this study, we compared the clinical outcomes, including corneal changes of flanged IOL fixation with IOL exchange and repositioned dislocated IOL for the treatment of IOL dislocation.

## Methods

This retrospective, consecutive, comparative interventional case series was conducted at the Nune Eye Hospital, South Korea, after approval of the Institutional Review Board of Nune Eye Hospital (IRB No. 230207-01), and all investigations adhered to the tenets of the Declaration of Helsinki. All study participants provided an informed consent. The medical records of all consecutive patients who underwent flanged IOL fixation combined with 25-gauge pars plana vitrectomy for the treatment of IOL dislocation in the Nune Eye Hospital from March 2019 to February 2022 were reviewed. The exclusion criteria included any other retinal diseases (retinal detachment, age-related macular degeneration, diabetic retinopathy, and retinal vein occlusion), corneal diseases (corneal opacity, corneal laceration, and corneal dystrophy), and other ocular diseases that can affect visual acuity (glaucoma and traumatic optic neuropathy).

### Surgical techniques

All surgeries were performed by a single surgeon (JRD). Using the CONSTELLATION Vision System (Alcon Laboratories, Inc., Duluth, GA, USA), all patients underwent 25-gauge pars plana vitrectomy. After vitrectomy, the reusability of the dislocated IOL was evaluated intraoperatively. If the dislocated IOL was one-piece or if a deformity was present, including IOL optics opacification, IOL optics damage, IOL haptic curvature deformation, or optic–haptic junction in a dislocated three-piece IOL, the dislocated IOL was removed via corneal incision and flanged IOL fixation with a new three-piece IOL (exchanged IOL fixation group) was performed, as previously described by Yamane et al.^7^ Briefly, after insertion of the new three-piece IOL (Tecnis ZA9003; Abbott Medical Optics Inc., Santa Ana, CA, USA) into the anterior chamber, two transconjunctival angled sclerotomies were performed 2 mm from the limbus at 4 and 10 o’clock using a 30-gauge thin-wall needle (TSK ultrathin wall needle; Tochigi Seiko, Tochigi, Japan). Two haptics of the IOL were docked into the 30-gauge needle lumen using retinal forceps in the anterior chamber. After extraction of the haptic out of the conjunctiva, the haptic ends were cauterized to make a flange through ophthalmic cautery.

**Figure 1:**
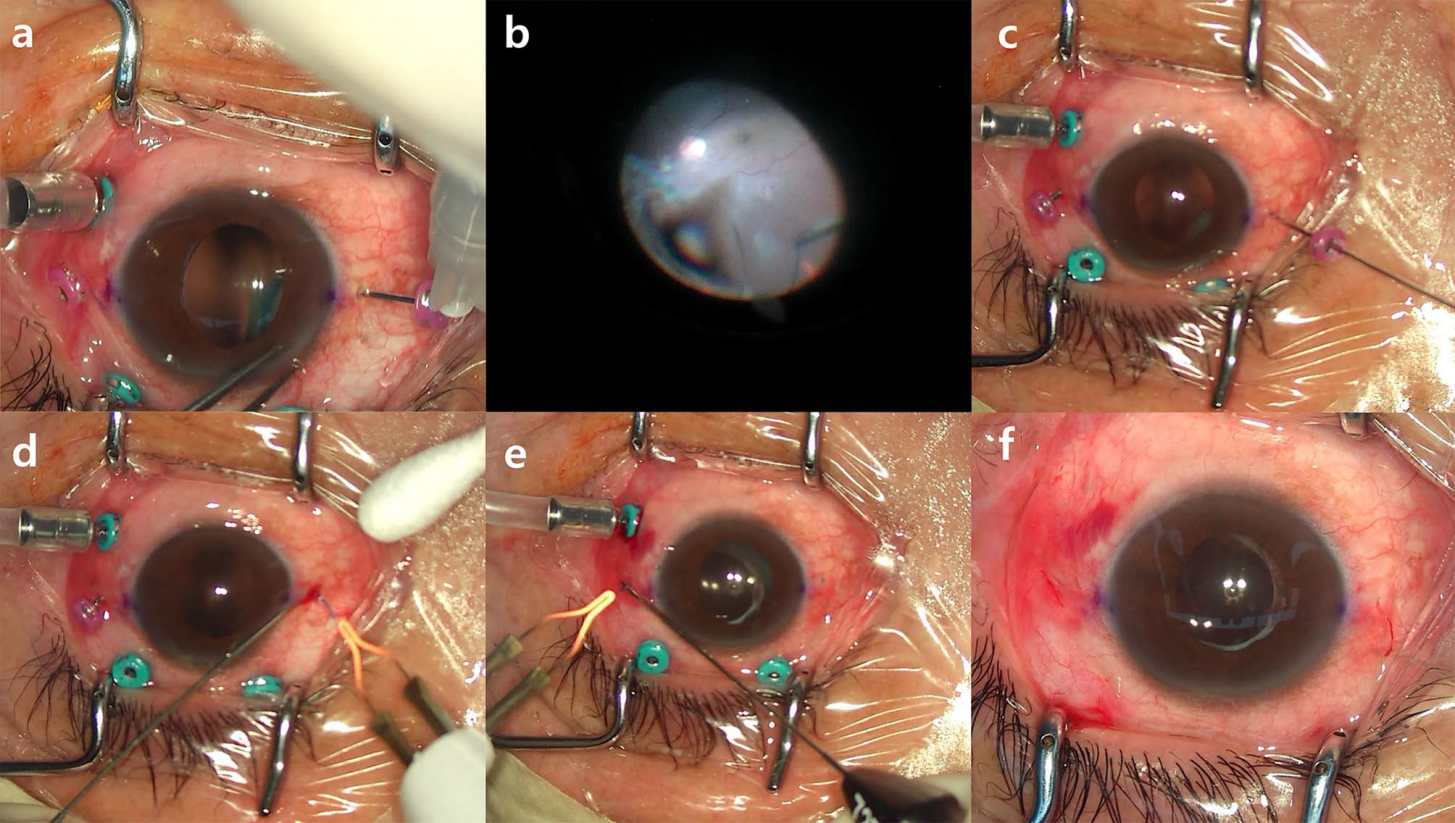
Surgical steps of flanged intraocular lens (IOL) fixation utilizing the dislocated three-piece IOL. (**a**) Two transconjunctival angled sclerotomies were performed 2 mm from the limbus at 3 and 9 o’clock using a 27-gauge trocar. (**b**) After verifying that the structure of the dislocated IOL was intact, the end of the dislocated IOL haptic was gently grasped using a 27-gauge retinal forceps in the vitreous cavity. (**c**) The haptic of the dislocated IOL was extracted from the conjunctiva using retinal forceps. (**d**) The haptic ends were cauterized to make a flange using an ophthalmic cautery. (**e**) The procedure was repeated for the second haptic of the dislocated IOL. (**f**) After adjusting the IOL centration, the second flanged haptic was placed into the sclera.

If the structures of the dislocated three-piece IOL were intact, flanged IOL fixation was performed using the dislocated IOL (repositioned IOL fixation group). For this technique, a 27-gauge trocar was used as previously described by Walsh et al.^9^ (Fig. 1). Briefly, two transconjunctival angled sclerotomies were performed 2 mm from the limbus at 3 and 9 o’clock using 27-gauge trocars. The end of the dislocated IOL haptic was gently grasped using 27-gauge retinal forceps in the vitreous cavity. The haptic ends were cauterized to make a flange using ophthalmic cautery after extracting the haptic from the conjunctiva using retinal forceps. During surgery, corneal incision was not required.

### Measurement of clinical outcomes

All patients underwent comprehensive ophthalmic examinations, including measurement of best-corrected visual acuity (BCVA) using the Snellen chart, slit-lamp examination, intraocular pressure (IOP), fundus examination, and spectral domain optical coherence tomography. Using a noncontact specular microscope (NSP-990, Konan, Japan), ECD was measured. Additionally, corneal astigmatism was assessed via autorefractor/keratometer (ARK-700A, Nidek, Japan). Changes in corneal astigmatism between baseline and at 1, 6, and 12 months postoperatively were analyzed, and keratometric data were converted into J0 and J45 values via power vector analysis^10^. In the power vector analysis, J0 and J45 indicates vertical/horizontal and oblique astigmatic changes, respectively. A positive J0 indicates a change toward against-the-rule (ATR) astigmatism, whereas a negative J0 indicates a change toward with-the-rule astigmatism. The IOL position was measured via swept-source anterior segment optical coherence tomography (CASIA2; Tomey Corp., Nagoya, Japan) after pupil dilation. Furthermore, the IOL position was evaluated in terms of horizontal and vertical axes in the IOL scan measurement mode via a three-dimensional analysis, as previously described^11^.

### Statistical analyses

All statistical analyses were conducted using SPSS version 18.0 for Windows (SPSS., Chicago, IL, USA). To compare numeric and categorical data, independent *t*-tests and chi-square tests were employed, respectively, between the groups. BCVA was converted to logarithm of minimum angle of resolution (logMAR) values for statistical analysis. During the follow-up period, differences in the changes in BCVA, ECD, and corneal astigmatism were compared using the Bonferroni method. Statistical significance was set at p < 0.05.

## Result

This study included 51 eyes of patients in the exchanged IOL fixation group (n = 51) and 38 eyes of patients in the repositioned IOL fixation group (n = 33). No significant difference was observed between the groups in terms of age, sex distribution, onset of IOL dislocation after previous cataract surgery, preoperative BCVA, IOP, axial length, and white-to-white distance (Table 1). The preoperative corneal astigmatism in the exchanged and repositioned IOL fixation groups were 1.25 ± 1.32 Diopter (D) (P > 0.05) and 1.28 ± 0.92 D (P > 0.05), respectively.

**Table 1:** Baseline characteristics of patients who underwent exchanged and repositioned intraocular lens (IOL) fixation for IOL dislocation.

	Exchanged IOL group (n = 51)	Repositioned IOL group (n = 38)	P value
Age (mean ± SD, years)	64.98 ± 10.9	63.10 ± 11.6	0.751^a^
Sex (male/female)	27/24	20/18	0.977^b^
BCVA (mean ± SD, logMAR)	0.62 ± 0.55	0.59 ± 0.64	0.946^a^
Intraocular pressure (mean ± SD, mmHg)	16.44 ± 5.7	16.30 ± 4.9	0.118^a^
Axial length (mean ± SD, mm)	24.82 ± 3.6	24.24 ± 1.8	0.863^a^
White-to-white (mean ± SD, mm)	11.96 ± 0.5	11.90 ± 0.7	0.357^a^
Onset from previous cataract surgery (mean ± SD, months)	100.8 ± 97.1	98.6 ± 94.9	0.916^a^
Endothelial cell density (cells/mm^2^)	2581.5 ± 466.6	2499.2 ± 192.1	0.447^a^
Corneal astigmatism (mean ± SD, Diopter)	1.25 ± 1.32	1.28 ± 0.92	0.917^a^

BCVA = best-corrected visual acuity; IOL = intraocular lens; logMAR = logarithm of the minimum angle of resolution; SD = standard deviation.

^a^P values were determined using a two-tailed, unpaired, two-sample *t*-test.

^b^P values were obtained using the chi-square test.

### Visual acuities

**Figure 2:**
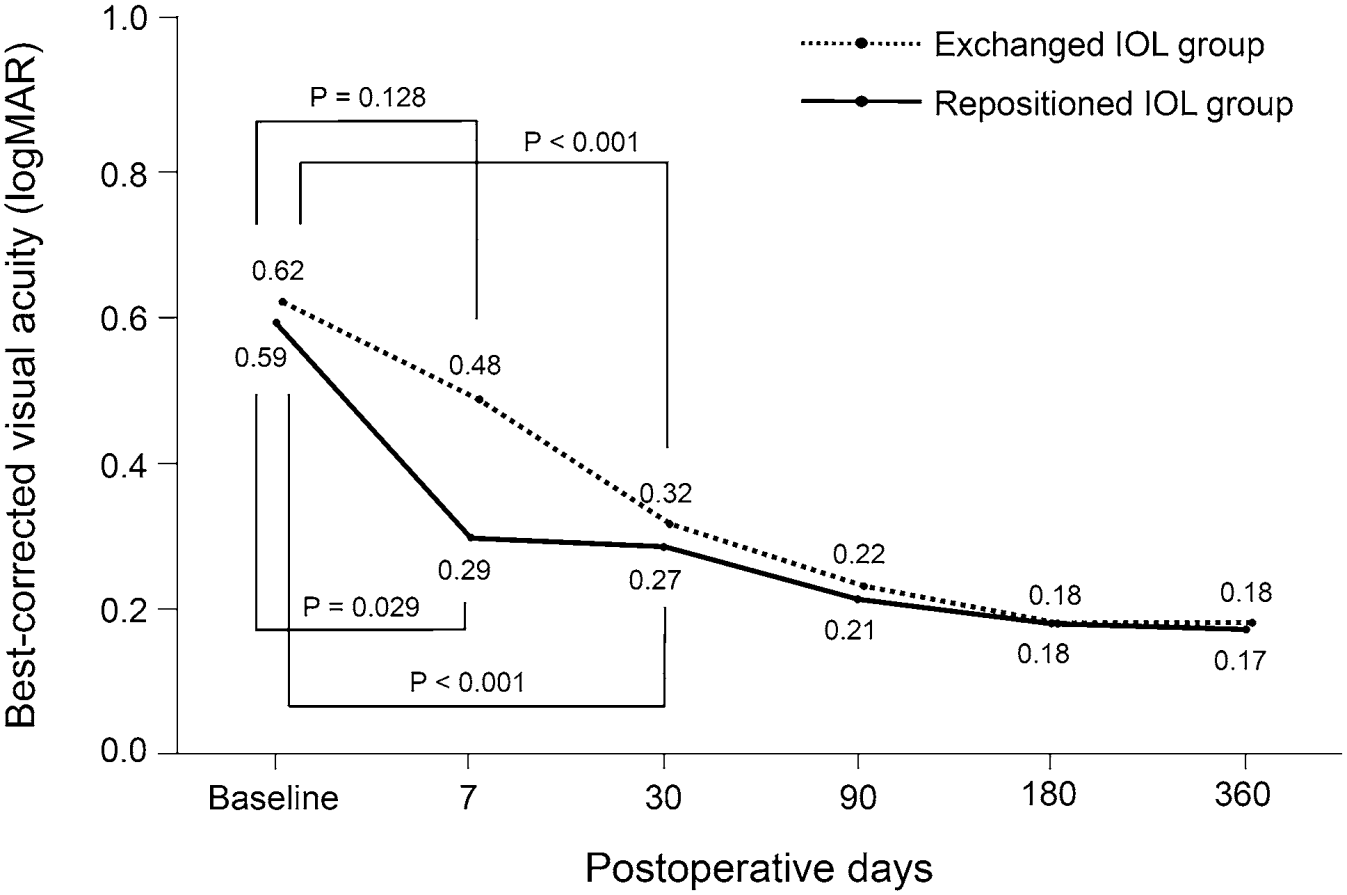
Changes in the postoperative best-corrected visual acuity (BCVA) after flanged IOL fixation with new IOL exchange and repositioned dislocated IOL in patients with IOL dislocation. The BCVA considerably improved at 1, 3, 6, and 12 months postoperatively in both the exchanged (P < 0.001 at all four time points) and repositioned (P < 0.001 at all four time points) IOL fixation groups compared with the preoperative BCVA. At 1 week postoperatively, the BCVA remarkably improved compared with the preoperative BCVA (P = 0.029). However, in the exchanged IOL fixation group, the BCVA at 1 week postoperatively did not differ from the preoperative BCVA (P > 0.05).

The BCVA at 12 months postoperatively was 0.18 ± 0.11 logMAR (Snellen 20/30) in the exchanged IOL fixation group and 0.17 ± 0.09 logMAR (Snellen 20/30) in the repositioned IOL fixation group (Fig. 2), indicating significant improvements compared with the preoperative BCVA (P < 0.001, and P < 0.001, respectively). In the exchanged IOL fixation group, the BCVA at 1 week postoperatively was 0.48 ± 0.41 logMAR (Snellen 20/60) and did not differ from the preoperative BCVA (0.62 ± 0.44 logMAR [Snellen 20/83]) (P > 0.05). However, in the repositioned IOL fixation group, the BCVA at 1 week postoperatively was 0.29 ± 0.27 logMAR (Snellen 20/39), indicating a significant improvement compared with the preoperative BCVA (0.59 ± 0.64 logMAR [Snellen 20/78]) (P = 0.029). The BCVA significantly improved at 1, 3, 6, and 12 months postoperatively compared with the preoperative BCVA in the exchanged (P < 0.001 at all four time points) and repositioned (P < 0.001 at all four time points) IOL fixation groups. Moreover, BCVA did not differ between the groups at 1, 3, 6, and 12 months postoperatively (P > 0.05).

### Changes in the corneal endothelial cell density and corneal astigmatism

The preoperative corneal ECD in the exchanged and repositioned IOL fixation were 2581.5 ± 466.6 cells/mm^2^ and 2499.2 ± 192.1 cells/mm^2^, respectively. No difference was observed between the groups (P > 0.05). However, the mean corneal ECD decreased more in the exchanged IOL fixation group (207.8 ± 209.5) than in the repositioned IOL fixation group (71.1 ± 74.8) (P = 0.007). The rate of ECD decrease in the group with exchanged and repositioned IOL fixation were 8.04% and 2.84%, respectively.

**Figure 3:**
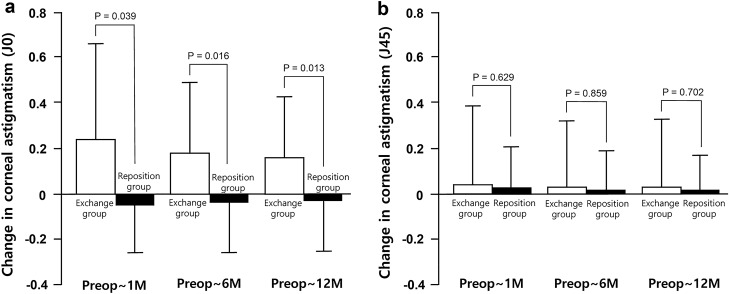
Comparison of the changes in vertical/horizontal corneal astigmatism (J0) and oblique corneal astigmatism (J45) between the exchanged and the repositioned IOL fixation group. (**a**) The mean J0 value in the exchanged IOL fixation group showed a more positive value than the repositioned IOL fixation group at postoperative 1, 6, and 12 months. (**b**) No differences were noted in the mean J45 values between the groups throughout the follow-up period.

Figure 3 presents the J0 and J45 values of corneal astigmatism in the exchanged and repositioned IOL fixation groups. The mean J0 value was more positive (ATR shift) in the exchanged IOL fixation group than in the repositioned IOL fixation group at 1, 6, and 12 months postoperatively (P = 0.039, 0.016, and 0.013, respectively). No between-group differences were noted in the mean J45 values throughout the follow-up period (all P > 0.05).

### Position of the IOL

The horizontal and vertical IOL tilts were 5.11° ± 2.45° and 3.26° ± 2.23° in the exchanged IOL fixation group and 4.83° ± 1.56° and 3.43° ± 3.12° in the repositioned IOL fixation group, respectively (Table 2). No significant difference was observed in the horizontal and vertical tilt between the groups (P > 0.05 and P > 0.05, respectively).

**Table 2:** Comparison of intraocular lens (IOL) tilt and decentration between the exchanged and repositioned flanged IOL fixation groups at 12 months after surgery.

	Exchanged IOL group (n = 51)	Repositioned IOL group (n = 38)	P value
Horizontal tilt (mean ± SD, °)	5.11 ± 2.45	4.83 ± 1.56	0.397
Vertical tilt (mean ± SD, °)	3.26 ± 2.23	3.43 ± 3.12	0.156
Horizontal decentration (mean ± SD, mm)	0.44 ± 0.13	0.43 ± 0.17	0.534
Vertical decentration (mean ± SD, mm)	0.36 ± 0.17	0.38 ± 0.21	0.646

IOL = intraocular lens; SD = standard deviation.

P values were determined using a two-tailed, unpaired, two-sample *t*-test.

IOL decentration was also compared by dividing the groups into horizontal and vertical axes. The horizontal and vertical IOL decentrations were 0.44 ± 0.13 mm and 0.36 ± 0.17 mm, respectively, in the exchanged IOL fixation group and 0.43 ± 0.17 mm and 0.38 ± 0.21 mm, respectively, in the repositioned IOL fixation group. No difference was observed in these values between the groups (P > 0.05 and P > 0.05, respectively).

### Postoperative complications

Table 3 presents the early postoperative complications that occurred within 1 month after surgery and late postoperative complications that occurred between 1 and 12 months after surgery. The incidence of early postoperative complications, including vitreous hemorrhage, hypotony, and IOP elevation, did not differ between the exchanged and repositioned IOL fixation groups (P > 0.05). However, corneal edema at 1 week postoperatively was more frequent in the exchanged IOL fixation group than in the repositioned IOL fixation group (P = 0.007). Late postoperative complication, including cystoid macular edema (CME), pupillary optic capture, redislocation of IOL, and corneal decompensation, did not differ between the groups (P > 0.05).

**Table 3:** Postoperative complications in the exchanged and repositioned intraocular lens fixation groups.

	Exchanged IOL group (n = 51)	Repositioned IOL group (n = 38)	P value
Early complications			
Corneal edema at 1 week postoperatively, n (%)	14 (27.5)	2 (5.3)	0.007
Vitreous hemorrhage, n (%)	2 (3.9)	1 (2.6)	0.739
Hypotony, n (%)	2 (3.9)	3 (7.9)	0.421
IOP elevation, n (%)	2 (3.9)	1 (2.6)	0.593
Late complications			
Cystoid macular edema, n (%)	4 (7.8)	2 (5.3)	0.631
Pupillary optic capture of the IOL, n (%)	2 (3.9)	2 (5.3)	0.763
Redislocation of the IOL, n (%)	0 (0)	1 (2.6)	0.244
Corneal decompensation, n (%)	1 (2.0)	0 (0)	0.385

IOL = intraocular lens; IOP = intraocular pressure.

P values were determined using the chi-square test.

## Discussion

This retrospective study aimed to compare the clinical and anatomical outcomes of flanged IOL fixation using either a repositioned dislocated IOL or a new IOL exchange after removal of the dislocated IOL in patients with IOL dislocation. The clinical outcomes were evaluated on the basis of visual acuity, ECD, corneal astigmatism, and occurrence of complications.

BCVA significantly improved at 1 month postoperatively compared with the preoperative BCVA in both surgical techniques, which was consistently maintained for 12 months. The mean postoperative BCVA of our study was similar to that of a previous study^8,12^. However, at 1 week postoperatively, the exchanged IOL fixation group did not exhibit improved BCVA similar to that in the previous study^8^, whereas the repositioned IOL fixation group had improved BCVA compared with that at baseline. This difference between the groups may be attributed to postoperative corneal edema at 1 week after surgery. A previous study reported that corneal edema following surgery decreased visual acuity, which improved within 1 month postoperatively^13^. In our study, corneal edema was significantly higher in the exchanged IOL fixation group than in the repositioned IOL fixation group at 1 week postoperatively, and most cases of corneal edema improved at 1 month postoperatively in both surgical techniques.

The decrease in postoperative ECD was considerably lesser in the repositioned IOL fixation group than in the exchanged IOL fixation group. There could be several reasons why postoperative corneal edema and ECD decrease were more obvious in the exchanged IOL fixation group. Exchanged IOL fixation techniques involve corneal incision, dislocated IOL removal, and a new IOL insertion through the corneal wound. During these procedures, corneal endothelial cell damage is inevitable, particularly when removing the three-piece IOL with long and rigid haptics. Conversely, in repositioned IOL fixation, the haptic end of the IOL was grasped using membrane forceps in the vitreous cavity with a wide-angle retina viewing system, distant from the iris and corneal endothelium. As a result, there was no need for an iris retractor, corneal incision, or corneal endothelial touch during the entire surgical procedure. Amon et al.^14^ reported that the scleral incision group had less endothelial cell loss than the clear corneal incision group in the cataract surgery. Similarly, in the case of exchanged IOL fixation, the scleral incision may be more beneficial in minimizing ECD decrease than the corneal incision.

Our study revealed that postoperative ATR astigmatic changes occurred in the exchanged IOL fixation group. This change may have occurred because we performed a 3–6-mm corneal incision superiorly to remove the dislocated IOL and insert the new IOL. Conversely, in the repositioned IOL fixation group, there was less astigmatic change compared with the exchanged IOL fixation group during the follow-up, possibly due to the absence of any corneal incision.

A previous study reported a higher incidence of postoperative CME in the exchanged IOL group than in the repositioned IOL group in patients with sutured scleral fixation^15^. This study suggested that this difference may be attributed to subclinical inflammation resulting from prolonged operating time. However, in our study, no significant difference was noted in the incidence of postoperative CME between the groups. This might be attributed to the fact that the operating time of flanged IOL fixation was less than half that of the sutured scleral fixation.

In the repositioned IOL fixation group, there was one case of redislocation after surgery, whereas redislocation did not occur in the exchanged IOL group; however, the difference between the groups was not statistically significant. IOL redislocation that occurred in the repositioned IOL fixation group was caused by the breakage of the optic–haptic junction at 1 week postoperatively. IOL fixed to the sclera using the flanged IOL fixation technique would apply greater tensile force on the haptic or optic–haptic junction compared with IOL using sutured scleral fixation. Additionally, the haptics of the dislocated IOL could undergo deformation due to the extended contraction of the capsular bag^16^. Therefore, even if a minor damage to the haptic or optic–haptic junction or optic is detected in the dislocated IOL, it should be exchanged with a new IOL instead of reusing it. Furthermore, when reusing the dislocated IOL, it is important to minimize manipulation of the haptic or optic–haptic junction of the dislocated IOL during surgery and gently manipulate it to minimize IOL damage. Additionally, the type of three-piece IOL should also be considered when deciding on the flanged IOL fixation with repositioned dislocated IOL. In south Korea, only four types of three-piece IOLs are commonly used: ZA9003 (Abbott Medical Optics Inc., Santa Ana, CA, USA), MA60AC (Alcon Laboratories, Fort Worth, TX), AR40e (Abbott Medical Optics Inc., Santa Ana, CA, USA), and PC-60AD (Hoya, Tokyo, Japan). Among these IOLs, PC-60AD is not suitable for this technique due to the bulging haptic end.

This study has several limitations, including a small sample size and relatively short postoperative follow-up period. We compared the clinical outcomes between the groups for only 1 year. However, several studies have reported that complications, including IOL redislocation, may occur after more than 5 years of sutured scleral fixation^17,18^. Thus, a longer follow-up monitoring of the cohort is warranted.

Previous studies have reported that corneal ECD decreased to 6.0–20.0% after cataract surgery and could not be regenerated^19,20^. Thus, patients with IOL dislocation who undergo cataract surgery may be relatively vulnerable to postoperative corneal complications, including corneal decompensation. In conclusion, flanged IOL fixation using the dislocated IOL was an effective and safe technique for minimizing corneal endothelial damage and astigmatic changes in a patient with three-piece IOL dislocation. However, it is important to thoroughly assess the dislocated IOL deformity before and during surgery and gently manipulate it to minimize IOL damage.

## Data Availability

The datasets used and/or analyzed during the current study available from the corresponding author on reasonable request.
