# Publisher Correction: Repositioned versus exchanged flanged intraocular lens fixation for intraocular lens dislocation

**DOI:** 10.1038/s41598-024-58105-8

**Published:** 2024-03-27

**Authors:** Yong Koo Kang, Dong Ho Park, Gahyung Ryu, Hong Kyun Kim, Dong Hyun Kim, Jae Rock Do

**Affiliations:** 1 Department of Ophthalmology, School of Medicine, Kyungpook National University, Kyungpook National University Hospital, 130 Dongdeok-Ro, Jung-Gu, Daegu, 41944 Republic of Korea; 2 Nune Eye Hospital, Seoul, Republic of Korea

Correction to: *Scientific Reports* 10.1038/s41598-024-54694-6, published online 14 March 2024

The Funding section in the original version of this Article was omitted. The Funding section now reads:

"DHP is financially supported by the Basic Science Research Program of the National Research Foundation of Korea (NRF), funded by the Ministry of Science and ICT (MSIT) (2019R1A2C1084371); the Information Technology Research Center (ITRC) support program funded by MSIT and supervised by the Institute of Information and Communications Technology Planning & Evaluation (IITP) (IITP-2023-2020-0-01808); the Korea Drug Development Fund (KDDF) funded by the MSIT, Ministry of Trade, Industry, and Energy, and Ministry of Health and Welfare (MOHW) (RS-2021-DD120784 (HN21C0923000021)); and the Korea Health Technology R&D Project through the Korea Health Industry Development Institute (KHIDI), funded by the MOHW (HR22C1832)."

The original Article has been corrected.

